# Climacteric symptoms are related to thyroid status in euthyroid menopausal women

**DOI:** 10.1007/s40618-019-01078-7

**Published:** 2019-08-07

**Authors:** R. Slopien, M. Owecki, A. Slopien, G. Bala, B. Meczekalski

**Affiliations:** 1 Department of Gynecological Endocrinology, Poznan University of Medical Sciences, Poznan, Poland; 2 Department of Public Health, Poznan University of Medical Sciences, Poznan, Poland; 3 Department of Child and Adolescent Psychiatry, Poznan University of Medical Sciences, Poznan, Poland

**Keywords:** Menopause, Thyroid, Thyroxine

## Abstract

**Background:**

Climacteric symptoms are a variety of disturbing complaints occurring during menopausal transition, many of which may be influenced by hormonal abnormalities other than related to sex steroids.

**Aim of the study:**

In this study, we investigated the association between the intensity of climacteric symptoms measured with the Kupperman index and a thyroid status.

**Material and methods:**

We evaluated by measuring serum thyrotropin (TSH), and free thyroxine (fT4) 202 euthyroid women admitted to the Department of Gynecological Endocrinology, Poznan University of Medical Sciences because of climacteric symptoms. Patients were both in perimenopause (n = 74) and postmenopause (n = 128), with no history of thyroid disorders.

**Results:**

Results presented as the mean value and standard deviation were as follows: age 54.2 ± 4.9 years, BMI 26.8 ± 4.6 kg/m^2^, Kupperman index 26 ± 13.1 points, TSH 2.4 ± 2.6 mU/l, fT4 1.2 ± 0.37 ng/dl. We observed a negative correlation between fT4 and the time since the last menses (*R* = − 0.38; *p* = 0.02) as well as between serum TSH concentration and sweating (*R* = − 0.18; *p* = 0.03), general weakness (*R* = − 0.17; *p* = 0.03), and palpitation (*R* = − 0.18; *p* = 0.02) and a positive correlation between fT4 and nervousness (*R* = 0.34; *p* = 0.007) and palpitations (*R* = 0.25; *p* = 0.04). In the perimenopausal subgroup, there was a positive correlation between fT4 and general weakness (*R* = 0.42; *p* = 0.03), palpitations (*R* = 0.50; *p* = 0.009), and paresthesia (*R* = 0.46; *p* = 0.01). In the postmenopausal subgroup, there was a negative correlation between TSH and sweating (*R* = − 0.21; *p* = 0.03).

**Conclusions:**

Menopausal symptoms are related to thyroid status in euthyroid menopausal women.

## Introduction

Climacteric symptoms are the primary complaint of women seeking medical treatment during their transition to menopause [1]. These symptoms not only impair quality of life [2] but also are an independent risk factor of cardiovascular disease [3] and osteoporosis [4]. The most common among complaints are vasomotor symptoms (hot flashes, sweating, palpitations, and headaches) but can include many others, such as insomnia, nervousness, low mood, vertigo, general weakness, arthralgia, paresthesia, and low libido [5–7]. The occurrence of climacteric symptoms in the western population varies between 50% [8] and more than 80% [9] of perimenopausal and postmenopausal women.

The main cause of climacteric symptoms is a sudden decrease in serum levels of 17β-estradiol which leads to an impairment of brain cell survival, metabolism, synaptic plasticity, and cerebral blood flow [10]. The serotonergic, noradrenergic, cholinergic, and GABA-ergic neurotransmitter systems are all relevant to the etiology of climacteric symptoms when impaired [11]. Among the risk factors for vasomotor symptoms, serum estrone/17β-estradiol ratio [12], obesity (BMI equal or higher than 31 kg/m^2^) [6], smoking [13], and Afro–American ethnicity [14] are also documented.

Thyroid gland activity is age dependent [15]. TSH serum concentrations rise with age in iodine-sufficient areas [15]. Older age is related to a higher frequency of hypothyroidism [16]. Routine screening for thyroid disease is recommended in menopausal women [17] as symptoms of thyroid disease can mimic those of menopause [18]. Some typical symptoms of hypothyroidism can often be mistaken for climacteric symptoms, such as poor memory, slower thinking, tiredness, and muscle cramps. Symptoms of hyperthyroidism, such as anxiety, sweating, palpitations, and insomnia can also be mistaken for those of menopause. It has been demonstrated that thyroid hormones influence brain function through similar mechanisms to estrogen: cell metabolism, gene expression, inter-cell signal transmission, and modulating the synthesis of enzymes needed for neurotransmitter production [19]. Similarly to estrogens, thyroid hormones modulate the action of noradrenergic, serotoninergic and GABA-ergic systems in the brain [20].

On the foundation of these similarities, we ask if there exists an association between thyroid status and the prevalence and severity of climacteric symptoms in euthyroid menopausal women. As of yet, no studies have been published on the subject. Therefore, the purpose of this study was to investigate the possible associations between menopausal symptoms and thyroid status.

## Materials and methods

In this retrospective study, we examined 202 women aged 42–65 years admitted to the Department of Gynecological Endocrinology, Poznań University of Medical Sciences, complaining of climacteric symptoms. Menopausal status of all study participants was confirmed by the presence of climacteric symptoms not explained by other illness, and exclusion of other hormonal disturbances. The exclusion criteria were: known thyroid disease, any other chronic diseases, and use of menopausal replacement therapy or other medications or supplements influencing menopausal symptoms.

Patients form the study pool (total group) were divided into two subgroups: perimenopausal group consisting of 74 women still menstruating or whose elapsed time since last menses was not longer than 12 months, and postmenopausal group consisting of 128 women who were at least 1 year after menopause.

Height and body weight of all patients were measured, and body mass index (BMI) calculated. The intensity of climacteric symptoms was evaluated using the Kupperman index [21]. In all study subjects, fasting serum thyrotropin (TSH), free thyroxine (fT4), folliculotropin (FSH), lutropin (LH), 17β-estradiol, prolactin (PRL), and total testosterone levels were measured with the use of chemiluminescence immunoassay (Roche Diagnostics, Mannheim, Germany). Intra-assay and inter-assay coefficients of variation (CV) ranges were 1.2–3.3% and 2.0–5.6%, respectively. Fasting dehydroepiandrosterone sulfate (DHEA-S) levels were measured using radioimmunoassay (Diagnostic Products Corporation, Los Angeles, CA, USA), intra-assay CV and inter-assay CV ranges were 5.1% and 11%, respectively.

Clinical and hormonal characteristics of the study participants were expressed as the mean ± standard deviation (SD). The Mann–Whitney test was used to evaluate differences between the study subgroups, while Spearman’s test was used to evaluate correlation between variables. All statistical analyses were conducted using SPSS ver. 15.0 (SPSS Inc., Chicago, IL, USA). A *p* value less than 0.05 was considered statistically significant.

The study protocol was approved by the Ethics Committee, Poznań University of Medical Sciences, and financed by the State Committee for Scientific Research (Project No: 50305-01109136-12261-08039).

## Results

Clinical and hormonal characteristics of both study subgroups are presented in Table 1. The frequency of climacteric symptoms is shown in Table 2. In women who were still menstruating, the mean serum TSH concentration was 2.2 ± 1.8 mU/l and the mean serum ft4 concentration was 1.1 ± 0.22 ng/dl. In postmenopausal women, the mean serum TSH concentration was 2.1 ± 1.3 mU/l, and the mean serum fT4 concentration was 1.3 ± 0.43 ng/dl. The difference between groups was not statistically significant. There was a correlation between fT4 and the time since last menstrual flow (*R* = − 0.38; *p* = 0.028) for the total group. When considering the study population as a whole, there was a negative correlation between serum TSH concentration and sweating (*R* = − 0.18; *p* = 0.03), general weakness (*R* = − 0.17; *p* = 0.03), and palpitations (*R* = − 0.18; *p* = 0.02). For the same group, a positive correlation was observed between fT4 and nervousness (*R* = 0.34; *p* = 0.007), and palpitations (*R* = 0.25; *p* = 0.04). In the premenopausal group, a positive correlation was observed between fT4 and general weakness (*R* = 0.42; *p* = 0.03), palpitation (*R* = 0.50; *p* = 0.009), and paresthesia (*R* = 0.46; *p* = 0.01). In the postmenopausal group, a negative correlation was observed between TSH and sweating (*R* = − 0.21; *p* = 0.03). There was no correlation between serum TSH concentration and: fT4, age, BMI, Kupperman index value, or other clinical and hormonal parameters.

**Table 1:** Clinical and hormonal characteristics of the total group, perimenopausal group and postmenopausal group (mean value ± SD) Mann–Whitney test

Parameter	Total group	Perimenopausal group	Postmenopausal group	*p*
Age (years)	54.2 ± 4.9	52.8 ± 3.6	56 ± 4.5	0.000000
Time since last menstruation (months/years)	3.9 ± 4.6 years	3.6 ± 2.8 months	5.6 ± 4.6 years	0.000043
BMI (kg/m^2^)	26.8 ± 4.6	26.6 ± 4.4	26.8 ± 5.3	0.708770
Kupperman index	26 ± 13.1	32.6 ± 13.5	25.4 ± 12.8	0.337729
TSH (mU/l)	2.4 ± 2.6	2.52 ± 2.7	3.9 ± 2.5	0.918623
fT4 (ng/dl)	1.2 ± 0.37	1.1 ± 0.2	1.3 ± 0.4	0.131593
FSH (IU/l)	67.1 ± 35.8	52.8 ± 40.8	75.5 ± 26.7	0.000005
LH (IU/l)	34.9 ± 17.5	32.6 ± 21.9	36.2 ± 14.1	0.103898
E2 (pg/ml)	48,1 ± 97.5	84.1 ± 126.6	26.9 ± 67.5	0.000001
PRL (ng/ml)	11.7 ± 5.7	15.9 ± 12.6	12.6 ± 8.2	0.021901
Total testosterone (ng/ml)	0.28 ± 0.2	0.3 ± 0.18	0.27 ± 0.2	0.078730
SHBG (nmol/l)	59.6 ± 37.8	57.2 ± 31.4	60.8 ± 40.4	0.573227
DHEAS (μg/dl)	1.4 ± 0.8	1.51 ± 0.9	1.27 ± 0.74	0.101973

**Table 2:** The frequency of climacteric symptoms in the total, perimenopausal and postmenpausal group (Mann–Whitney test)

Kupperman climacteric symptom—number of patients (%)	Total group	Perimenopausal group	Postmenopausal group	*p*
Hot flashes	158 (78.2%)	54 (72.9%)	104 (81.1%)	0.780333
Sweating	169 (83.7%)	63 (85.1%)	106 (82.7%)	0.112691
Insomnia	136 (67.3%)	49 (66.2%)	87 (67.9)	0.818202
Nervousness	160 (79.2%)	59 (79.7%)	101 (78.8%)	0.372916
Low mood	130 (64.4%)	50 (67.5%)	80 (62.4%)	0.686575
Vertigo	103 (50.9%)	39 (52.7%)	64 (49.9%)	0.174995
General weakness	128 (63.4%)	54 (72.9%)	74 (57.7%)	0.002401
Arthralgia	140 (69.3%)	48 (64.8%)	92 (71.8%)	0.200057
Headaches	115 (56.9%)	45 (60.8%)	70 (54.6%)	0.347866
Palpitations	132 (65.3%)	46 (62.1%)	86 (67.1%)	0.901629
Paresthesia	127 (62.9%)	46 (62.1%)	81 (63.2%)	0.746092

## Discussion

In our study population, all women were euthyroid when recruited, with no statistically significant difference in mean TSH or mean fT4 between perimenopausal and postmenopausal subgroups. The serum hormone levels for participants were distributed within the accepted limits of normal; however, a negative correlation between fT4 and time since last menstruation was observed. This may indicate the impact of menopausal status on thyroid function. Studies have reported an increase in the level of serum TSH and a decline in serum fT4 with age [22, 23] which leads to the rise in frequency of hypothyroidism in older women. In contrast, most studies report no effect of menopause on the levels of thyroid hormone, and a negative effect on thyroid binding globulin (TBG) [22, 23].

To our knowledge, there are no studies examining the possible relationship between serum TSH or fT4 with the severity of climacteric symptoms as measured using the Kupperman index. When considering climacteric symptoms and their intensity as a whole, we found no correlation between serum TSH and fT4 concentrations and overall Kupperman index value. When separating the index into its component elements, however, we found several correlations between individual climacteric symptoms and serum thyroid hormones.

In this study, we observed that serum TSH concentration correlates negatively with sweating, and general weakness. This is in line with the most typical symptoms of subclinical hyperthyroidism, the incidence of which increases in concordance with decreasing serum TSH concentration. Of note is that in this study, we did not observe overt hyperthyroidism per se in the patient sample, despite this correlation. Similarly, we found that serum TSH correlated negatively with palpitations, whereas fT4 correlated positively with palpitations. Here again, a similar pattern can be seen: in perimenopausal women, it is sufficient that only a statistical tendency in TSH and fT4 towards their respective hyperthyroid deviations correlates with clinical symptoms very specific for hyperthyroidism. This observation raises three subsequent issues. Firstly, the condition that we call “pre-hyperthyroid status,” i.e., TSH in the lower range of normal accompanied by normal or high-normal fT4, is perhaps more common in perimenopause than widely assumed as it usually goes unreported since TSH remains in the normal reference ranges. Secondly, as a consequence, this may lead to greater intensity of certain menopausal symptoms, which are frequently resistant to treatment in clinical practice. Thirdly, if this is the case, many menopausal women who are “pre-hyperthyroid status” would benefit from low-dose anti-thyroid medication as it might reduce the intensity of their symptoms. Under these circumstances, we postulate that in the near future, and following broader discussions, the reference ranges for these hormones should be adjusted accordingly.

A positive correlation between nervousness and fT4 found in the whole group is, to our knowledge, the first such report in menopausal women. Similar observations, in fact, are reported in the psychiatric literature. For example, the relationship between nervousness and fT4 was observed in studies on neuroticism, panic disorder, and anxiety. Frey et al. reported an association between neuroticism and low TSH levels in 121 healthy subject (NEO Five-Factor Inventory) [24]. Van de Ven et al. also reported an association between neuroticism and thyroid peroxidase antibodies (anti-TPOAb) [25]. Both Bensenor et al. [26] and Fardella et al. [27] described an association between subclinical hyperthyroidism and panic disorder. Panicker et al. reported on the trend towards an inverse relationship between TSH serum concentration and anxiety [28].

We demonstrated a positive correlation between serum fT4 and paresthesia in premenopausal women. This finding adds weight to the association between subjective feelings and thyroid status, even in the absence of a classic thyroid disorder. It appears higher free thyroxine serum concentration in the presence of dynamic estrogen and progesterone fluctuations may cause increased feelings of discomfort within the peripheral neuromuscular system. The exact molecular mechanism behind this interaction, however, remains unclear.

It appears that even minimal thyroid status shifts while euthyroid predisposes to changes in psychological wellbeing. This phenomenon would normally not be observed; however, when amplified by the presence of severe estrogen fluctuations, it becomes clinically relevant. We think that the increased influence of thyroid status on nervousness in menopausal women is directly triggered by loss of estrogen. Interestingly, in spite of the relationship between the levels of FT4 and nervousness, we did not demonstrate a negative correlation between TSH and nervousness. The absence of this latter association is somewhat confusing as compared with the finding of a positive correlation between FT4 and nervousness, but we think it might be related to some subclinical dysregulation of pituitary secretion in the perimenopausal period. It may be presumed that, being exposed to significant decrease of estrogen and thus producing large amounts of gonadotropins, the pituitary may concomitantly secrete slightly higher amounts of TSH, that are subtle and therefore not detectable under usual circumstances. This increase of pituitary activity might reduce the possible negative association between TSH and nervousness, even under the condition of periodic but subtle elevation of thyroid hormones (as we show for FT4 in this study). The association would obviously appear in hyperthyroidism, where pathological mechanisms are sufficient to completely suppress TSH secretion even in the presence of the overall increased pituitary activity. However, as long as hyperthyroidism does not develop, this association is absent.

Beyond those already mentioned, no additional relationships were found between thyroid status and climacteric symptoms. This requires some discussion presented below.

Unlike in the papers previously cited, we found no correlation between depressive mood and thyroid hormones. Similar results are reported in the literature, where no relationship between depression and thyroid status is observed [29–31]. The literature, however, also maintains that both clinical and subclinical hypothyroidism cause mild to severe depression [32–34] and that the presence of anti-TPOAb is related to depression and postpartum depression even in the absence of hypothyroidism [35, 36]. Furthermore, the relation between thyroid status and insomnia was reported by various authors. Both hyperthyroidism and hypothyroidism cause sleep problems such as difficulties initiating sleep, maintaining sleep, and slow wave sleep disturbances [37]. Thyrotropin-releasing hormone (TRH) injection reduces both non-rapid eye movement sleep and sleep efficiency, increasing wakefulness [38]. We did not observe any significant disturbances with regard to sleep, but this is likely since the patient sample in this study was euthyroid, and such subtle changes of fT4 were not sufficient to cause appreciable sleep disturbances.

We found no correlation between thyroid status in menopausal women and headache. Limited information exists on this point, most notably by Blanchin et al. who report that anti-TPOAb binds to human cerebellar astrocytes in patients with Hashimoto’s encephalopathy [39].

We found no correlation between thyroid status in menopausal women and vertigo. Several observational and retrospective studies have reported a relationship between thyroid autoimmunity and vestibular disorders. In one study, patients with Meniere disease had higher titers of thyroid autoantibodies when compared with healthy controls [40]. In another, patients with benign paroxysmal positional vertigo were shown to have higher serum TSH and thyroid autoantibody levels compared to healthy controls [41]. Madugno et al. suggest a possible mechanism in that diffusion of immune complexes into the inner ear and the potential change of endolymph composition causes increased mechanical stimulation of receptors and provokes positional vertigo [42].

When considering arthralgia, a litany of rheumatic manifestations has been described in association with autoimmune thyroiditis. Benhatchi et al. report detecting positive ANA antibodies in 45% of patients with autoimmune thyroiditis compared with 14.7% of healthy controls. In the same study, frequency of arthralgia and arthritis in patients with autoimmune thyroiditis was significantly higher than in healthy controls (50.0% vs. 20.6% and 23.7% vs. 2.9%) [43].

We did not observe any correlation between serum TSH or fT4 concentration and BMI. This is likely a result of the relatively low number of participants in the study. In large population studies on euthyroid individuals, serum TSH is usually positively associated with body weight and BMI [44].

Interestingly, we found no correlation between thyroid status and serum 17β-estradiol concentration. Such a correlation was previously reported in studies on estrogen replacement therapy in women with hypothyroidism treated with thyroxine. Estrogen replacement therapy causes changes in fT4 and TSH by increasing the binding of thyroxine to thyroxine-binding globulin [45].

In this first report on the association between climacteric symptoms and thyroid status, we concluded that nervousness in menopausal women is related to thyroid hormone status. We present these results as an additional argument in favor of thyroid hormone screening in menopausal women.
